# Tracking an occluded visual target with sequences of saccades

**DOI:** 10.1167/jov.22.1.9

**Published:** 2022-01-18

**Authors:** Tuisku Tammi, Jami Pekkanen, Samuel Tuhkanen, Lauri Oksama, Otto Lappi

**Affiliations:** 1 Cognitive Science, University of Helsinki, Helsinki, Finland; 2 National Defence University, Finland; 3 Human Performance Division, Finnish Defence Research Agency, Finland; 4 Traffic Research Unit, University of Helsinki, Helsinki, Finland

**Keywords:** object tracking, prediction, eye movements, smooth pursuit, saccades

## Abstract

Gaze behavior during visual tracking consists of a combination of pursuit and saccadic movements. When the tracked object is intermittently occluded, the role of smooth pursuit is reduced, with a corresponding increase in the role of saccades. However, studies of visual tracking during occlusion have focused only on the first few saccades, usually with occlusion periods of less than 1 second in duration. We investigated tracking on a circular trajectory with random occlusions and found that an occluded object can be tracked reliably for up to several seconds with mainly anticipatory saccades and very little smooth pursuit. Furthermore, we investigated the accumulation of uncertainty in prediction and found that prediction errors seem to accumulate faster when an absolute reference frame is not available during tracking. We suggest that the observed saccadic tracking reflects the use of a time-based internal estimate of object position that is anchored to the environment via fixations.

## Introduction

Visual tracking of moving targets is achieved by a combination of smooth pursuit eye movements and saccades (Dodge, 1903). The relative contributions from these two types of oculomotor behaviors to tracking depend on factors such as target trajectory and speed (Buizza & Schmid, 1986), target motion predictability (Hayhoe et al., 2012; Kowler, 1989), the presence and predictability of visual occlusions (Bennett & Barnes, 2003; Orban de Xivry et al., 2008), and task strategy (Wang & Kowler, 2021). Tracking objects in natural contexts usually happens in a cluttered scene, where the object of interest is intermittently blocked behind an occlusion and then reappears—and our tracking systems seem to cope with these occlusions quite well. By what mechanisms this is achieved remains an important question of vision science. Over the decades, work on natural gaze behaviors, including visual tracking, has revealed highly systematic patterns in humans and other animals (Hayhoe & Ballard, 2005; Hayhoe, 2017; Land, 2019; Lappi, 2016). The consistency in tracking behavior across individuals, and even species, makes visual tracking an attractive model behavior for experimental and modelling studies of perceptual-motor coordination both in the laboratory and in the wild (Krauzlis, 2004; Lisberger et al., 1987; Lisberger, 2015).

The need for accurate tracking is often justified in terms of facilitating the foveal (high resolution) analysis of visual detail, achieved by smooth pursuit stabilizing the retinal image (by matching gaze velocity to target velocity) and saccades then compensating for the positional error that accumulates due to gaze lagging behind the target (less than unity pursuit gain; Becker & Fuchs, 1985). Smooth pursuit velocity has consistently been shown to decrease significantly when a tracked object disappears, and saccades overshooting the object can be observed (Bennett & Barnes, 2003; Becker & Fuchs, 1985; Orban de Xivry et al., 2008). This kind of behavior is typically interpreted as a design shortcoming in the tracking system, with a decay in the memory used to drive pursuit, and/or impotence of internal representations in driving smooth pursuit in the absence of retinal feedback (Barnes, 2008; Becker & Fuchs, 1985). In other words, while the processes leading to the anticipatory saccade can be used to program saccade targets, they are unable to “drive” pursuit based on a constantly updated (dynamic) target position estimate.

However, when considering more complex, natural interactions with dynamic scenes, there are also considerations that can be raised against this type of “architectural” explanation for the reduction of pursuit and the emergence of saccades. For example, when there are multiple objects of interest, at most one object can be tracked foveally at a time while the rest have to (and can) be tracked peripherally (Cavanagh & Alvarez, 2005). Indeed, in multi-object tracking, the best solution may be to keep gaze fixed in space and track all objects peripherally (Fehd & Seiffert, 2010; but see also Hyönä et al., 2019). Therefore, continually aligning gaze with the target(s) is not always tantamount to successful tracking. Even in specifically designed tasks where the observer is instructed to track a single target, it is not obvious what the most desirable way of tracking for a given display is when an element of occlusion is introduced—during occlusion, there is nothing to be kept in foveal vision, so it is not apparent why the visual system should necessarily aim to maintain pursuit.

It has been suggested that, during occlusion, rather than trying to minimize the current position error, the tracking system's goal would be to minimize the position error at the time of target reappearance (Orban de Xivry et al., 2009). But does this actually predict tracking to occur predominantly by smooth pursuit or saccades during occlusion? Intuitively, pursuit would seem to be best, as accurate pursuit would ensure that gaze is at the correct location at all times (unless the reappearance location of the object is known—in that case, a simple anticipatory saccade to that location will suffice). This intuition is what gives initial plausibility to the idea that any drop in pursuit (gain) and the emergence of (compensatory) saccades would reflect some in-built limitation of the tracking system in generating pursuit movements.

In this laboratory eye-tracking study, we study visual tracking of an intermittently occluded object with a nonlinear trajectory. Because occlusion durations used in previous studies have been either predictable (fixed duration or represented by a physical occluder; e.g., Mrotek & Soechting, 2007; Bennett et al., 2007) or relatively short (e.g. Orban de Xivry et al., 2008; Bogadhi et al., 2013), we explore whether tracking can be maintained accurately for extended occlusion periods, up to several seconds, of unknown duration (varying randomly between trials). Further, we examine the features of gaze behavior over the course of the occlusion; that is, the relative contribution of smooth pursuit versus saccadic tracking. We interpret our findings in terms of a dynamic internal estimate of motion trajectory (cf. Orban de Xivry et al., 2008), but with special consideration given to the coordinate systems used to maintain this estimate.

We presented participants with a visual tracking task with five main characteristics (1–5 below):

(1) Nonlinear (circular) target motion
(2) Intermittent occlusion of the target
(3) Unpredictable occlusion duration

Our design had two novel features:

(4) A discrimination task at the end of the occlusion, which could only be performed foveally or parafoveally. This was to incorporate overt tracking into the task design, rather than rely on instruction.
(5) Occlusion durations that were long enough—up to 3 seconds. This was to explore extended pursuit and/or sequences of saccades (whichever we would observe).

Properties 1 through 3 are similar to previous studies by Orban de Xivry et al. (2008, 2009). The use of nonlinear target motion with intermittent visual occlusions is motivated by the fact that when the object disappears, anticipating its trajectory with gaze cannot be achieved by simply continuing to track in the direction, and at the speed of the object, at the moment of disappearance. Rather, to be successful, visual anticipation during the occlusion must be adapted to the (predictable) changes in object motion. This means that the brain probably has to retain a dynamic representation of object motion that is used to guide the eye. Consequently, movements of the eye can be used to probe this internal representation and its properties.

Note that the unpredictable duration of the occlusion is critical to prevent a strategy where the reappearance location can be determined on the basis of a preview (the way that a car disappearing behind a truck can be predicted to reappear at the other end of the truck). With a predictable occlusion duration, a heuristic strategy of making a saccade to the other end of the occluder, and then waiting there for the target to arrive, can be used (Mrotek & Soechting, 2007). This would, of course, negate the opportunity of using gaze behavior as an index of a continually updated dynamic estimate of target position.

Especially for a task where participants are expected to track an occluded object that remains invisible for a longer duration of time, we felt that the motivation for accurate tracking should not rely merely on the instruction given. This is why we introduced a discrimination task in the design (fourth characteristic). We argue that using a discrimination task increases the validity of using overt gaze tracking during occlusion as an index of the underlying dynamic representation, and it should be used more in future studies (see, e.g., Bennett & Barnes, 2006, for similar motivators in tracking). Performance in the discrimination task also provides a measure for tracking accuracy, in addition to gaze position.

The discrimination task is crucial because it is possible to track targets without foveating them, and because during occlusion there is nothing to foveate—it may be argued that when the target disappears from view, the simple instruction to “track the object” becomes ambiguous. In contrast, we did not wish to make an explicit instruction to “track by visual pursuit,” as this could interfere with the natural gaze behavior of the participants. Instead, we structured the task to encourage pursuit, including a goal and reward structure (feedback). We hypothesized that this would challenge the participants’ tracking mechanisms to maintain accurate, continual tracking of (estimated) object position even when the object is not visible.

The fifth characteristic, to our knowledge, extends the duration of the occlusion period beyond those used in this type of paradigm before. Many studies of predictive saccade dynamics tend to focus only on the first few saccades; occlusions in circular or curved-path tracking studies have been at most one second (Orban de Xivry et al., 2008, 2009; Mrotek & Soechting, 2007). Longer occlusion periods allow us to raise various questions about gaze behavior during tracking. If we were to observe extended pursuit, then the commonly seen shift from smooth pursuit to saccades might partly be a matter of how the task goals are framed rather than inherent design limitations of the oculomotor system. In contrast, if tracking is observed to be predominantly saccadic (even beyond the first saccade or two), this will allow us to explore how extended multi-saccade sequences are guided by dynamic internal representations of target motion.

Thus, our analyses will cover three areas: first, seeing how the addition of the discrimination task affects the gaze behavior observed in prior research (Orban de Xivry et al., 2008, 2009); second, examining the gaze features observed over extended occlusion durations; and third, probing the properties of internal representations of object motion, namely how uncertainty in tracking develops over the course of the occlusion. Based on our findings, we will discuss the coordinate system (absolute/retinotopic) used to represent target dynamics, proposing that our findings, and previous results from similar tasks, could be more parsimoniously explained by using an allocentric reference frame than just by in-built physiological limitations of the smooth pursuit system. Experiments that could be done to test this (re)interpretation against the standard view are proposed.

## Methods

### Participants

A convenience sample of ten participants (8 females, 2 males, aged 21–40 years) was recruited from university mailing lists. Participants reported normal or corrected-to-normal visual acuity and no known conditions affecting eye movements. They were remunerated with activity vouchers (worth 10 euros) for their participation.

### Materials

Eye movements were recorded with a binocular, head-mounted Pupil Core eye-tracker (Pupil Labs UG {haftungsbeschränkt}, Berlin, Germany). The eye cameras recorded at 120 Hz at 640×480 pixel resolution while the forward-facing scene camera recorded at 60 Hz at 1,280×720 resolution. The open-source Pupil Capture software (https://github.com/pupil-labs/pupil) was used for recording and calibration. Four optical markers were placed on screen corners to allow for mapping gaze from the headset's forward-facing scene camera image to screen coordinates.

The experiment was presented on a LG OLED55C7V 55” screen with participants sitting at a fixed distance of 85 cm on a Playseat Evolution gaming chair (Playseat Evolution Alcantara, Playseats B.V., the Netherlands). The software for the tracking task was developed in-house and is available as open-source (https://github.com/jampekka/webtrajsim/tree/speedest18). All software ran on an HP ENVY Phoenix 860-081no (Intel Core i7-6700K CPU, NVIDIA GeForce GTX 980 TI GPU) desktop computer running a Debian GNU/Linux as the operating system.

### Design

**Figure 1.**
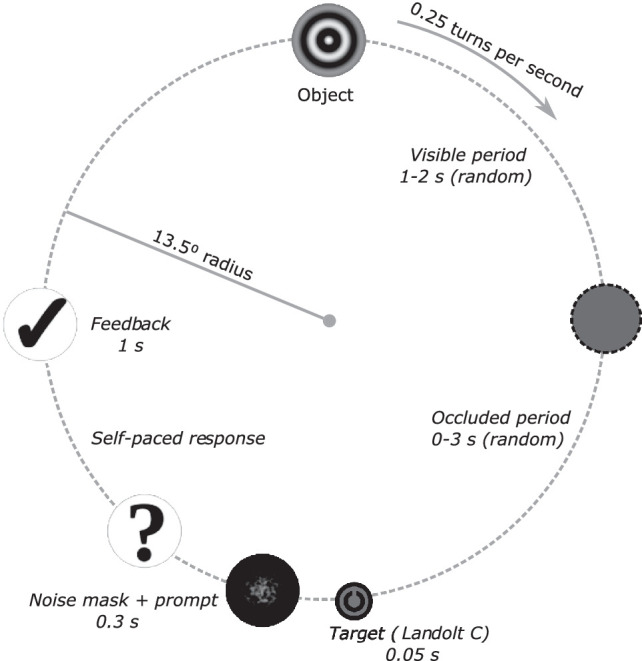
Characteristics of the trajectory and the events within a trial. The object moved clockwise at a constant rate of 0.25 turns per second, with a fixed radius of 13.5°, resulting in a vectorial velocity of 21°/s. After a period of visibility (randomly drawn from a uniform distribution between 1 and 2 seconds), the object disappeared (randomly drawn from a uniform distribution between 0 and 3 seconds). After the occlusion, the object reappeared with the target pattern (Landolt C) inside for 0.05 seconds, was masked, and the participant was prompted for a response. The participant then indicated which direction the letter C was facing (up, down, left, right), and received feedback for their response (for 1 second). The feedback period was immediately followed by the start of a new trial, that is, the appearance of the object to be tracked. Note that, although the experiment consisted of trials, the constant circular motion was not interrupted, even during the response and feedback periods.

The participants were presented with a small object on the screen, moving along a clockwise circular trajectory. Figure 1 illustrates the task design (and see https://zenodo.org/record/5468679 for a video of the task). The object moved at a constant rate (one full cycle in 4 seconds) with a fixed radius of 13.5°, with the circle center positioned at the center of the screen. In each trial of the main task, the object was visible for a random time in the range of 1 to 2 seconds, then disappeared for a random time between 0 and 3 seconds, and then reappeared with a Landolt C inside for 0.05 seconds. The participant's task was to report which direction the letter C was facing (up, down, left, right), using arrow keys on a standard keypad. Immediately after responding, the participant received visual feedback (correct/false), after which the next trial started without breaking the continuous circular motion.

There were 4 blocks of 30 trials of circular motion, the first block consisting of practice trials where the object was always visible; the other 3 blocks included intermittent occlusions. Additionally, in the beginning of the experiment, there were stationary practice trials, where participants fixated on a central fixation cross, and the letter C was presented at various distances from it (between 1.125° and 9°), facing up, down, left, or right. This task was included to make sure that participants understood the discrimination task, and to provide a control measure for accuracy in gaze position required for successful performance in the task (see the appendix for performance in the pretest trials). The full experiment also contained two other types of trajectories (linear horizontal motion and falling motion), the results of which are not reported here. The experiment in full took approximately 45 minutes to complete.

### Procedure

Before the experiment, participants gave their informed consent and completed a short background information form (age, gender, visual acuity, use of contact lenses). Participants were instructed to track the moving object closely and to perform the discrimination task as accurately as possible. Participants were then equipped with the eye tracking headset, the eye-tracker was adjusted and calibrated, and the participants proceeded to complete the experiment at their own pace. Calibrations of the eye-tracker were carried out between blocks. During the experiment, participants were free to revoke their participation at any point, or to take breaks between blocks.

### Data processing

Each gaze point was given a pupil detection confidence value between 0 and 1, based on the ratio of the detected pupil edge length and the fitted ellipse circumference. Data below the confidence level of 0.8 (only 0.5% of samples during the tracking task) were excluded from the analysis.

Gaze positions were estimated as visual angles by assuming an 80° horizontal field of view (FOV) for the monitor, that is, the visual angles were derived from screen pixel positions by a factor of 80/1920. This transformation approximately maps pixels to visual degrees for the circular path's radius given the seating and display setup.^1^ It should be noted that the true conversion from display positions to visual angles depends on the participants' head position and orientation, but for the sake of clarity, we opt to use this approximation.

As the participants’ heads were not fixed in relation to the screen, movements of the head caused the eye-in-head and gaze-on-screen coordinates to diverge. To compute the gaze-on-screen coordinates, head-to-screen position and orientation was estimated using an unscented Kalman smoother (UKS; Särkkä, 2013). The eye-tracker vendor's (Pupil Labs) solution is to compute a homography transformation independently for each frame using fiducial markers, but in our wide FOV setup, this tends to cause problematic high-frequency noise, especially when markers go outside the camera's FOV or are not recognized. Our smoothing uses the same fiducial markers, but by taking into account the whole time series, the filtering improves the head tracking performance and removes practically all of the high-frequency noise. Our UKS approach represents the forward facing camera's pose as position $\vec{p}=\left[x,y,z\right]$ relative to the screen center and rotation $\vec{R_{e}}=\left[yaw,pitch,roll\right]$ around the camera's optical center. The transition model assumes that variance increases by 0.01 screen heights per second per axis, and the rotation variance by 1 radian per second per axis. The observation model projects the marker corners using a pinhole camera model on the camera projection plane and assumes a spherical measurement error variance of 0.05 screen heights. The UKS solution maximizes the total likelihood of the camera orientation trajectory given these and the usual UKS assumptions and approximations. Means of the resulting pose distributions were used as the pose for further analyses.

**Figure 2.**
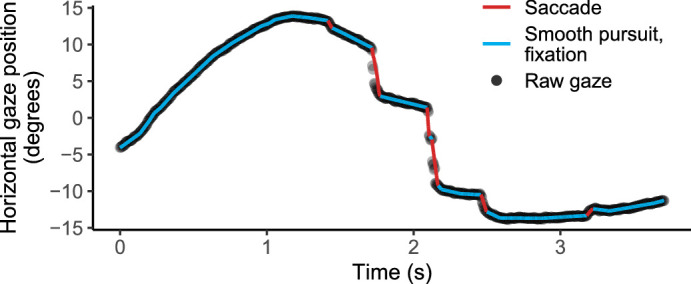
Example of gaze data classified into saccade and smooth pursuit/fixation segments. Raw gaze data shown as black dots. Lines indicate segmented saccades (red) and smooth pursuits or fixations (blue).

The gaze signal, in visual angles, was denoised, and saccade, pursuit, and fixation segments were detected using the naive segmented linear regression algorithm (Pekkanen & Lappi, 2017), with a hidden Markov model classifier to categorize segments. For the purposes of our analysis, pursuit and fixation classes were combined into one category. See Figure 2 for a time series of raw and segmented gaze signal.

For analyses conducted at the segment level, that is, where whole pursuit/fixation or saccade segments were classified according to the period (visible/occluded) they occurred in, segments that crossed both visible and occluded periods of a trial were classified into a third category. This was done to better examine the differences between the two periods of interest while avoiding splitting the segments.

Raw data are available at https://zenodo.org/record/5468679 and the code for data processing and analysis is available at https://github.com/ttammi/blindpursuit20.

## Results

**Figure 3.**
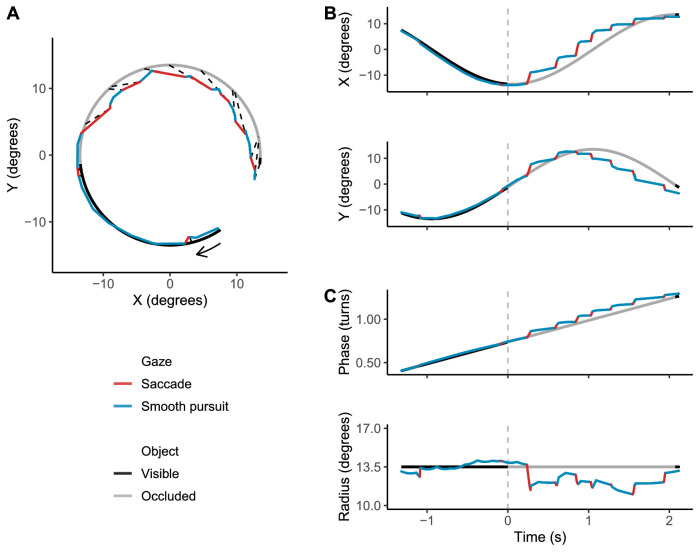
Example of gaze behavior and target object motion in a trial, illustrated in different coordinate systems. (A) Screen X-Y coordinates. Origin at the circle center. Black solid line: target object visible. Gray solid line: target object occluded. Arrow indicates the direction of motion (clockwise). Blue and red lines show gaze path, segmented into saccade (red) and smooth pursuit/fixation (blue). Dashed black lines are isochronic lines showing object and gaze positions at the timepoints of saccade launches and saccade landings. (B) Object and gaze motion decomposed into horizontal (X) and vertical (Y) components over time. (C) Phase and radius components of object and gaze positions over time. Occlusion onset at t = 0 marked by dashed grey line. Positive phase difference (gaze trace above object trace) indicates that gaze is ahead of the object, negative difference indicates lagging behind. The object moved at a constant rate of 0.25 turns per second and a fixed radius 13.5°. Note that when the object was visible, it was tracked mostly by smooth pursuit with few small saccades. When the object disappeared, the tracking became saccadic, interspersed by fixations or very low gain pursuit. These saccades tended to be anticipatory, clearly landing ahead of the object.

We investigated the gaze features in tracking an object on a circular trajectory with occasional occlusions. Figure 3 illustrates the gaze behavior in this task, showing smooth pursuits and saccades during a single trial. Figure 3 also shows the decomposition of gaze behavior relative to object motion in Cartesian (x-y) coordinates and polar (radius-phase) coordinates.

Polar coordinates are convenient in illustrating motion along a circular trajectory because they capture both the distance from the origin (radius, measured in degrees) and the angle on the trajectory (phase, measured in turns of the cycle). Specifically, the radius difference (gaze radius minus object radius) indicates whether gaze is located somewhere along the circular trajectory or inside/outside the circle, and the phase difference (gaze phase minus object phase) measures the timing accuracy of tracking—whether gaze is located ahead or behind the object.

The object was tracked closely during occlusion, the average total positional error (angular displacement between gaze position and target object position) being 5.91° at target reappearance (*SD* 4.92). Correspondingly, the average absolute phase difference was 0.07 turns (*SD* 0.07) and radius difference 2.07° (*SD* 1.87).

In the discrimination task, the participant-wise success rate ranged from 32% to 75%, with an overall rate of 57%. Generally, discrimination performance was better when gaze was close to target: for example, with position error at most 1.5°, the overall success rate was more than 90% (51% with greater position errors). Correspondingly, the average position errors at target reappearance were 4.39° (*SD* 4.19°) and 7.90° (*SD* 5.10°) for correct and false trials, respectively. Discrimination performance seemed to be slightly worse after longer occlusions: with occlusion duration at most 1 second, the overall success rate was 70%, whereas it was 49% for occlusions exceeding 1 second.

### Development of positional error over time

**Figure 4.**
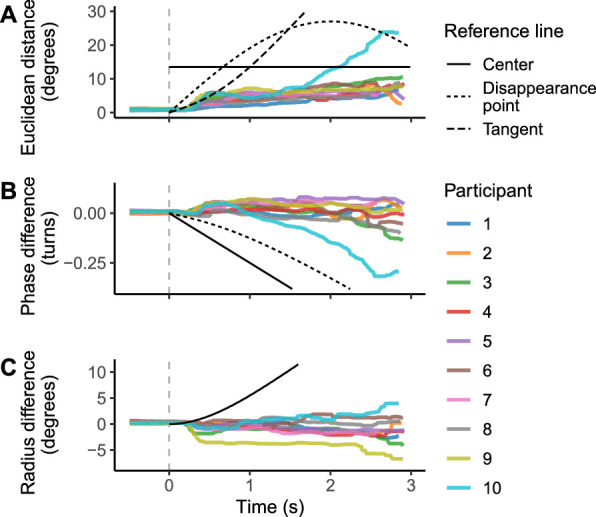
Participant-wise median gaze-to-target displacement (positional error) development over time, computed from linearly interpolated time series (60 data points per second). On average, gaze remained reasonably close to the actual target trajectory over a period of up to 3 seconds (the longest occlusions in this experiment), even if the tracking was mostly saccadic. Participant 10 deviated from others to some extent, owing to a tendency to glance at screen corners during the trials. (A) Absolute error magnitude in degrees. (B) Phase component. (C) Radial component. Occlusion onset at t = 0 marked by dashed grey line. Reference lines show, where appropriate, the positional error from hypothetical alternative gaze trajectories for comparison (see legend). Solid black line: gaze continuing along the tangent of object motion at disappearance. Dotted black line: gaze remaining at the disappearance point. Dashed black line: gaze shifting to the center of the circle/screen.

Throughout the course of the occlusion, gaze remained close to the target object for most participants. Both phase and radius difference remained quite unbiased over time (i.e., the difference was distributed around zero). This is illustrated in Figure 4 with time series of participant-wise medians of error magnitude (Euclidean distance, phase, and radius difference) during visible and occluded object tracking, referenced to the moment of target object disappearance.

We calculated hypothetical gaze trajectories based on alternative ways of tracking nonlinear motion, including continuing along a straight path tangential to the motion at disappearance, stopping at the disappearance point, or shifting to the center of the circle. The tangent trajectory would reflect the maintenance of speed information but no dynamic updating of direction, whereas stopping around the disappearance point would suggest that no information at all is retained. Shifting to the center, in contrast, would minimize the expected position error after the occlusion, given rough knowledge of the circle position but no dynamic estimation of the phase. These hypothetical trajectories are also illustrated in Figure 4. Clearly, none of these alternatives were at play here, but the circular path was extrapolated during occlusion, and positional errors remained modest for the majority of participants.

### Saccadic tracking during occlusion

**Figure A4.**
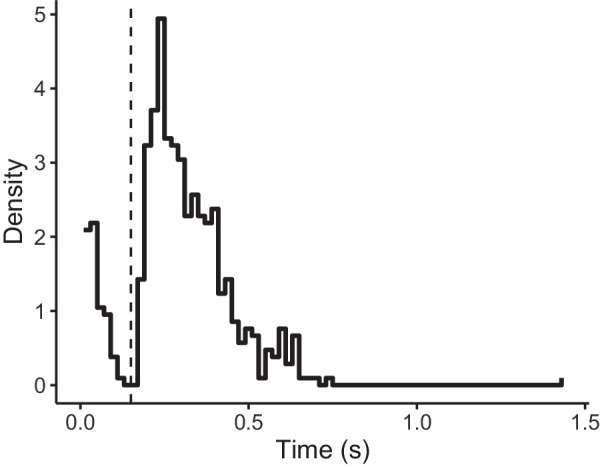
Latencies (launch times) of the first saccades after the occlusion begins at t = 0. Saccades occurring before 0.15 seconds (marked with dashed line) can be considered visually guided, that is, not yet reacting to the occluded target.

When the object was visible, it was tracked by smooth pursuit movements with some small-amplitude saccades (overall frequency 1.49 saccades/s and amplitude median 1.83°). When the object was occluded, we observed a change in gaze behavior: the trajectory was tracked mostly by saccadic eye movements–more saccades were made (2.88 saccades/s), with a median amplitude of 4.49°. Consistent with previous reports (Orban de Xivry et al., 2009), the typical reaction time to the disappearance of the object (i.e., the latency of the first saccade) was about 0.2 to 0.5 seconds (see Appendix Figure A4 for latency histogram).

**Figure 5.**
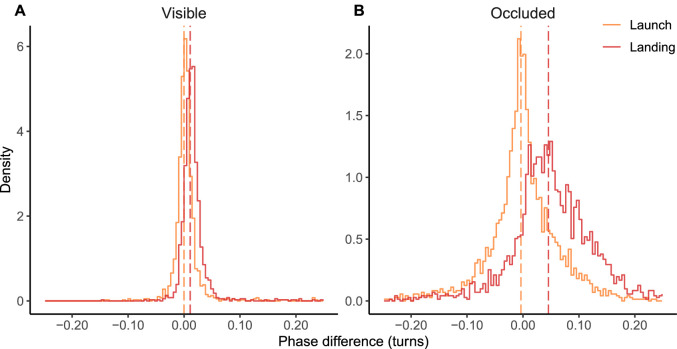
Distributions of phase difference of saccade launch and landing points. Positive phase difference indicates that gaze is ahead of the object. Dashed vertical lines indicate median phase differences. (A) saccades during visible period (launch median 0.00 turns, landing median 0.01 turns). (B) saccades made when target is occluded (launch median 0.00 turns, landing median 0.05 turns). When tracking the visible object, saccades launched and landed very close to the target, whereas when tracking an occluded target, the saccades tended to be launched at the target, with gaze landing well ahead and waiting there for the arriving target.

During occlusion, saccades were anticipatory: they were launched roughly at the object position and landed further ahead on the trajectory (see Figure 5). The absolute magnitude of phase difference was greater for landing than for launch points for all but one participant (binomial test, *p* = .021; overall medians of absolute phase difference were 0.03 turns and 0.05 turns for launch and landing points, respectively). In contrast, during visible periods, saccades were very small in terms of phase angle covered and were not predominantly either anticipatory or catching up (starting from behind and landing on the target, i.e., cancelling position error); medians of absolute phase difference were 0.01 turns for both launch and landing points. As can be seen in Figure 5, the phase lead of saccade landing points during occlusion was manyfold compared to landing points in visible periods (0.05 vs. 0.01 turns, respectively). It is noteworthy that a phase lead of this magnitude was not too much to impair discrimination performance; for example, the average position error at target reappearance in correct trials (4.39°) corresponds with a phase difference of 0.05 turns, assuming that gaze is positioned on the trajectory.

**Figure 6.**
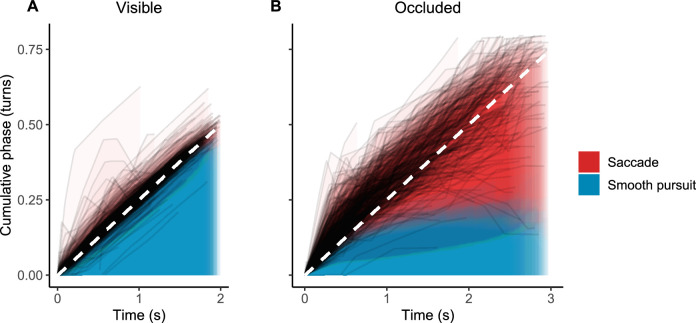
Gaze behavior was different depending on target visibility. Panels show cumulative phase angle covered by smooth pursuit (blue) and saccades (red) during visible (A) and occluded (B) object tracking. Note that visible periods lasted for 1 to 2 seconds and occluded periods for 0 to 3 seconds. Dashed white line shows the phase of the tracked object. When the target was visible, the phase was covered mostly by smooth pursuit. During occlusion, most of the phase angle change was covered by saccades, especially one second or more into the occlusion. Note also how the gaze tended to anticipate the target phase (gaze traces above the dashed line during occlusion). Trials from all ten participants are overlaid, showing a considerably consistent pattern across participants; see Appendix Figure A6 for a participant-wise figure.

To illustrate the change in behavior, we compared the phase angle covered by pursuits and saccades in visible and occluded periods. We did this by depicting the cumulative phase angle traveled along the circular trajectory (measured in turns) at each time point of the trials, for pursuits and saccades separately; see Figure 6. During periods when the tracked object was visible, the total phase angle covered was almost entirely by smooth pursuit (median 91% of total phase covered). During periods of occlusion, considerably less of the phase angle was covered by smooth pursuit (median 36%). This was remarkably consistent across our sample: for all ten participants, after 1.5 seconds of tracking (time chosen for a sufficient number of both visible and occluded data points), the median proportion of phase covered by pursuit was above 85% in the visible period and below 42% in the occluded period (binomial test *p* = .002). As can be seen in Figure 6, especially the saccades occurring shortly after the occlusion onset tended to move the gaze well ahead of the object trajectory.

**Figure 7.**
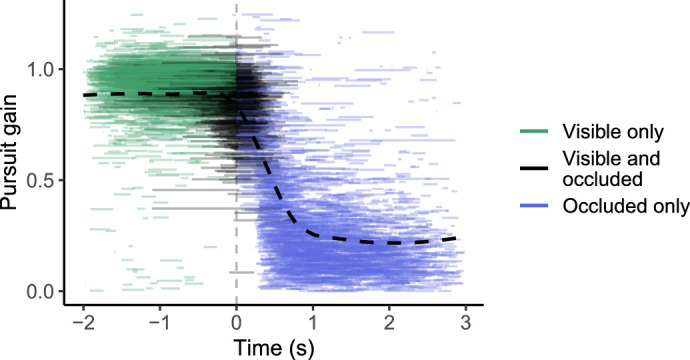
Pursuit gain in different periods of the trial, calculated from the phase component: the ratio of the change in gaze phase during the pursuit segment to the change in object phase in the same interval. Each line shows the beginning and ending times of a pursuit segment so that line length indicates segment duration. Colors denote visually-guided pursuits before the occlusion (green), segments spanning the occlusion starting point (black), and ”blind” pursuits when the target is occluded (blue). Occlusion onset at t = 0 marked by dashed grey line. Dashed black curve indicates loess regression fit.

The shift to saccadic tracking was clearly reflected in pursuit gain, which was close to one during visible periods and dropped considerably after the occlusion onset. This is illustrated in Figure 7, which shows the gain of pursuit segments during both visible and occluded periods. Corresponding to the observed reaction time of roughly 0.2 to 0.5 seconds to the object disappearance (i.e., latencies of first saccades after occlusion onset), pursuit gain dropped mostly during the first second of the occlusion, after which the tracking became predominantly saccadic.

### Uncertainty in internal estimates

**Figure 8.**
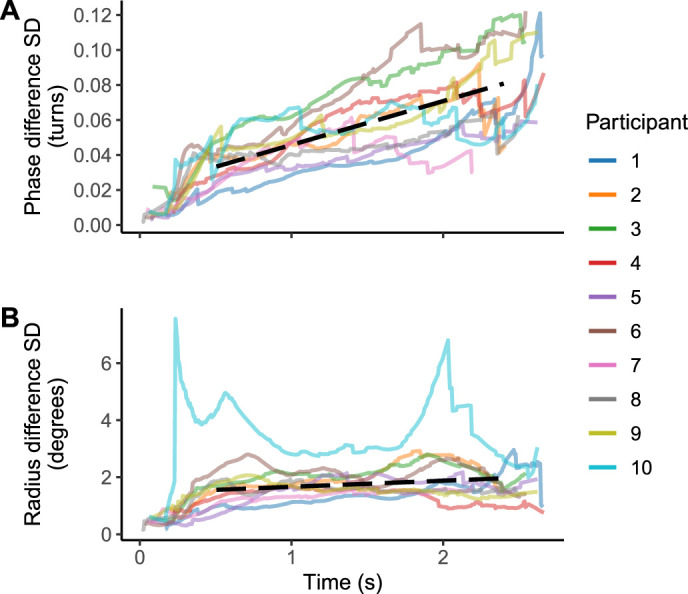
Participant-wise standard deviations of phase (A) and radius (B) difference over time, following the occlusion onset at t = 0. This indexes the accumulation of uncertainty of target position. Computed from linearly interpolated saccade launch time traces (500 data points per participant), including only time points where at least five traces were available. Decomposition of positional error into the radial and phase components suggests different profiles of uncertainty: the variability of gaze radius reached a plateau after 0.5 seconds, whereas the variability in phase—the current position of gaze on the circular trajectory - kept accumulating over time. Dashed black curves indicate regression lines (radius or phase difference *SD* as the dependent variable, time as the independent variable, participant 10 excluded; see the appendix for regression details). As a rough estimate, after the initial 0.5 seconds, the phase *SD* increased about 0.025 turns per second (marginal $R^{2}=0.35$), or if interpreted as time estimate, about 0.1 seconds per second. In contrast, the rate of change in radius *SD* was 0.21° per second (marginal $R^{2}=0.05$), with values remaining mostly below 2°.

To probe the accumulation of uncertainty in the internal target position estimate during the occlusion period, we investigated the development of the standard deviation of both radius difference and phase difference over time. The radius difference *SD* is linked to uncertainty in the estimated size or position of the circular trajectory, whereas the phase difference *SD* reflects uncertainty in time estimates, that is, how far along the trajectory the object has travelled. We used saccade launch points as a measure of the internal estimate, assuming that gaze was kept slightly ahead of the estimated target position and anticipatory saccades were made when the object was likely to reach the current gaze position. Figure 8 suggests that the positional error (*SD*) of phase may accumulate differently compared with that of the radius: Following an initial increase for roughly 0.5 s (i.e., when the saccadic tracking stabilized), the radius difference *SD* remained approximately constant, mostly below 2°. In contrast, the phase difference *SD* seemed to increase over time: as a rough estimate, after the initial 0.5 seconds, it increased at a rate of 0.025 turns per second, or if interpreted as time estimate, about 0.1 seconds per second.

## Discussion

We observed a clear difference in gaze behavior in occluded tracking of circular motion, compared to visually guided tracking: mainly saccades are used to cover the phase displacement for periods up to 3 seconds. More specifically, gaze behavior is characterized by a decline in smooth pursuit gain shortly after the occlusion begins, followed by a shift to saccadic tracking where consecutive anticipatory saccades land ahead of the actual (unobservable) object position. These observations are in line with previous findings about tracking circular motion with relatively similar characteristics of the trajectory (radius and object velocity; see Orban de Xivry et al., 2008, 2009). We extend these prior results by examining a longer period of occlusion, finding that the object can be tracked closely even during extended occlusions. Importantly, the tracking occurs by sequences of saccades even with the addition of a primary task that relies on analysis of fine texture in (para)foveal vision (to recognize the direction of a Landolt C at the end of randomised occlusion) and would incur a performance cost from retinal blurring during a fixation, therefore favoring pursuit tracking. Longer occlusion durations were linked to slightly higher position errors and worse discrimination performance, as could be expected. Interestingly, the magnitude of the phase lead created by most of the anticipatory saccades we observed corresponds with the average position error where the discrimination task could still be performed correctly. Additionally, even though the path (the shape of the circle) of the occluded object was tracked rather successfully, the uncertainty in phase (where on the circle the target will appear) seemed to accumulate over time.

Orban de Xivry et al. (2009) suggest that, for occluded targets, anticipatory saccades may emerge to minimize position error at target reappearance, rather than at any point during the occlusion. Moreover, the authors explain the occurrence of anticipatory saccades by arguing that the sensory costs related to visually guided tracking, namely, insufficient vision resulting from either position error or the execution of saccades, become insignificant during occlusions, which is why the goal during occluded tracking is not to minimize these costs. Although the brain is able to know where the occluded target is, this information is not fully reflected in the gaze position (Orban de Xivry et al., 2009). However, by using the pattern recognition task coupled with random-duration occlusions, we have added such costs: because the target might reappear at any point during the occlusion, gaze should not be too far away from the tracked object, and missing the target owing to saccadic suppression should be avoided. In other words, there should be a balance between executing saccades and the position error at each point. This is, in fact, a key motivation for using random occlusions, as it is not possible to know exactly when target reappearance will occur–the situation would be very different with occlusions of a known duration.

Future studies could examine the factors affecting the magnitudes and landing points of anticipatory saccades or, using a larger sample of participants, between-subject differences in gaze behaviors (such as the trade-offs involved in making many small saccades, in contrast to few large saccades). For example, a larger target might not require gaze to be as close to the object and could have an effect on the size of the lead created by anticipatory saccades. It should be noted that, in our study, the discrimination task served as a motivator to keep tracking the object, and we were not interested in discrimination performance or visual acuity per se, but this kind of task could provide interesting opportunities for studying dynamic visual acuity during occlusion, building on existing studies with visible targets (see, e.g., Palidis et al., 2017; Uchida et al., 2012).

Why pursuit velocity is not typically sustained in the absence of a visual stimulus is an old question, and the drop in pursuit gain is typically attributed to limitations of the oculomotor system (Becker & Fuchs, 1985; Kowler, 2011). A discrimination task such as ours would intuitively strongly favor tracking with smooth pursuit, if one assumes that i) the brain is able to establish a dynamic estimate (prediction) of object motion during visual pursuit and maintain it during the occlusion, and ii) this dynamic estimate can be used to endogenously “drive” smooth pursuit. The first assumption is supported by previous occluded tracking results (Orban de Xivry et al., 2008), as well as our observations of successful tracking during occlusions. In this framework, the shift to saccadic tracking would seem to reflect a shortcoming in the ocular system's ability to generate pursuit movements (i.e., violation of the second assumption), consistent with the conclusions in previous work on occluded tracking tasks. This interpretation is also commonly found in textbooks and models of pursuit, relying on retinal slip (Lencer et al., 2019). Essentially, the ocular system would be unable to engage in tracking by smooth pursuit in the absence of a visual target.

However, there is an alternative, or at least complementary, interpretation. This is suggested to us by the accumulation of variability in the time-dependent (phase) component while the variability of the fixed (radius) component of positional error stays relatively stable over time. Why would this difference be significant? During occlusion, there is no visual information available about the phase, that is, where on the circular trajectory the object is. This forces the estimate of tracked object position to rely entirely on a time-based internal estimate of target dynamics (an internal representation of the trajectory and object speed). However, if these dynamics are represented in world coordinates (where the trajectory is a simple circle) rather than retinotopic coordinates (where the path of the object is a complex shape because of the saccadic eye-movement behavior), then useful visual information *is* available during occlusion, on each fixation. During fixations, this visual information can be used to recalibrate the trajectory (center and radius) relative to the new post-saccadic gaze position, resulting in reduced uncertainty in radial position, but not phase. If this is the case, fixation might be a better means of visual sampling for the purposes of accurate calibration, compared with pursuit, which introduces retinal blur of the visual periphery (Schütz et al., 2007). If you consider, in contrast, maintaining an estimate of the tracked object's location under purely retinotopic tracking (where the internal dynamic model only maintains information about target position relative to the fovea), an extended sequence of saccades would be surprising. This is because each saccade would accumulate noise in the internal estimate (from amplitude and direction variability) that goes unchecked by visual feedback, in the absence of a visible object or other relevant visual input. The visual shifts of the stable visual periphery would be irrelevant if the object motion is represented relative to the fovea rather than to the stable visual scene.

So, under the standard interpretation, the functioning of the tracking system during occlusion appears compromised: “failing” to maintain pursuit and being forced to track with saccades. An alternative approach for developing models of visual tracking could build on the presumption that the tracking system is optimized for visual tracking that is not done in complete darkness (almost never the case in natural ecological conditions), but in the presence of a stable visual background (very often the case in natural ecological conditions), and has to be able to handle visual occlusions (implying that the system cannot rely on the position of the target pattern on the retina only). The change in gaze behavior observed in visual pursuit under occlusion might therefore reflect simply a change in the most reliable and useful information available: at object disappearance, object motion and position information is lost, and the tracking system reorganizes its sampling behavior to maximize intake of the next best, most useful, information to minimize prediction uncertainty, specifically, reference points for calibrating a trajectory estimate that is invariant with respect to eye movement (such as the screen frame). It should be noted that while, in our view, the observed features of saccadic tracking might imply the use of an allocentric frame of reference during occluded tracking, we are not asserting anything about the reference frame used during *visible* object tracking, as smooth pursuit tracking does not rule out the use of allocentric information (Fehd & Seiffert, 2010). Furthermore, we emphasize that we are not viewing smooth pursuit and saccade systems as two separate systems, as they are known to interact (Krauzlis, 2004; Lappi, 2016; Goettker & Gegenfurtner, 2021)—pursuits and saccades are simply the observed gaze features that are useful for characterizing gaze behavior in this task.

We propose that tracking during occlusion in our task—and similar tasks in the literature—might be based on a dynamic internal estimate of target motion that takes advantage of an allocentric frame of reference. If this is so, then saccadic tracking can be (re)interpreted as an effective means for extracting information from the environment to maintain the fixed constituents of such internal model. Even though it may seem self-evident that a simple trajectory such as a fixed circular shape can be tracked also during occlusion, it is unclear how a low radius error during saccadic tracking would be maintained if tracking was based on a retinotopic reference frame; in other words, accounting for the circular shape with respect to the environment is already committing to some kind of allocentric representation. Accordingly, we suggest that visual tracking models should incorporate a mechanism for maintaining the mapping between retinotopic and allocentric coordinates. Both alternatives—as far as we can tell—are consistent with the body of experimental evidence available. In the experiment by Orban de Xivry et al. (2008), tracking was performed in complete darkness (i.e., without any visual reference), and therefore does not leave room for our alternative interpretation, which is based on exploiting environmental cues. Nevertheless, we did observe a similar gaze pattern in the beginning of the occlusion as Orban de Xivry et al. For this reason, it would be intriguing to see whether the gaze patterns observed in our study would be replicated during extended occlusions in complete darkness.

Although our analysis was confined to within-trial accumulation of error, the learning process in this task could be further examined by analysing how the variation of phase error evolves across trials, reflecting development in the internal estimate. Principally, the different hypotheses (allocentric vs. retinotopic coordinates in tracking) would call for a formal specification that allows for predictions to be derived and tested on new data, making use of the potential manipulations outlined above. One key element in mapping out the plausibility of the allocentric hypothesis would be introducing variations in the key characteristics of the trajectory (radius, velocity)—in other words, the predictability of the trajectory—to probe their effects on tracking behavior. Another interesting aspect for future studies could be the role of hand–eye coordination in tracking under extended occlusions (in the dark), as manual tracking has been shown to promote smooth pursuit tracking even with unpredictable motion trajectories (Niehorster et al., 2015). Furthermore, adding an external temporal reference frame—such as a metronome—might facilitate the estimation of time, which should improve tracking accuracy by reducing phase uncertainty.

## Conclusion

We found a clear difference between gaze behavior in visually guided and occluded tracking of circular motion: during occlusion, most of the gaze displacement is made with saccades, but the occluded object can still be tracked successfully for several seconds. The participants did not maintain smooth pursuit to track the occluded object despite the use of a discrimination task. Based on these observations, as well as the accumulation of uncertainty in phase but not radius estimates, we discuss the reference frame used to maintain the dynamic internal estimate of target motion. We consider the effectiveness of saccadic tracking in extracting information from the environment to maintain the fixed constituents of the internal estimate, and suggest that the tracking system might use an allocentric frame of reference instead of a retinotopic one. In the future, formal models should be developed to test the plausibility of this interpretation.

## Figures and Tables

**Figure A1.**
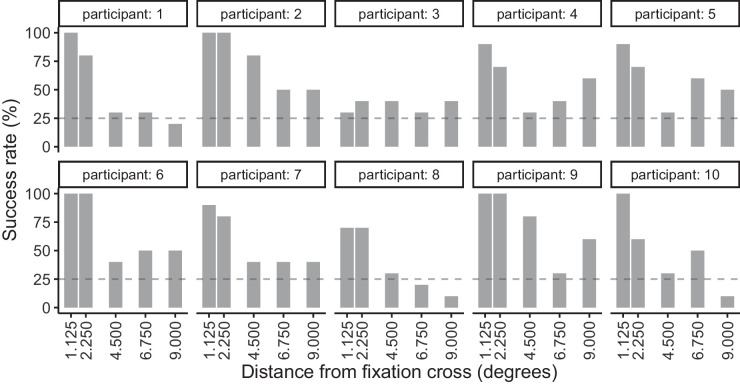
Participant-wise success rates by distance of target from fixation cross in pretest condition. There were a total of 50 trials, 10 per each distance. Dashed line indicates chance level (25%).

**Figure A2.**
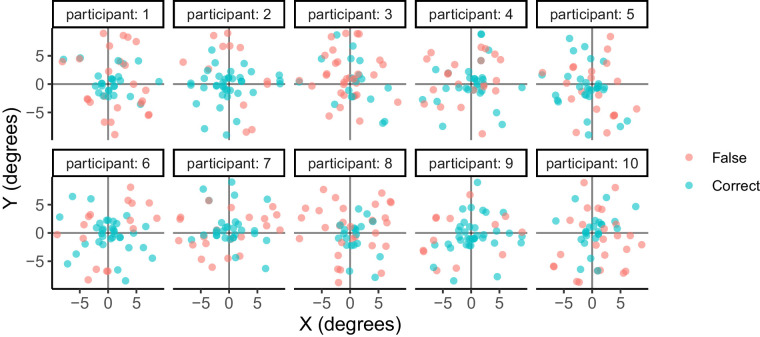
Participant-wise correct and false trials in the pretest condition by position, in degrees, relative to the fixation cross.

**Figure A3.**
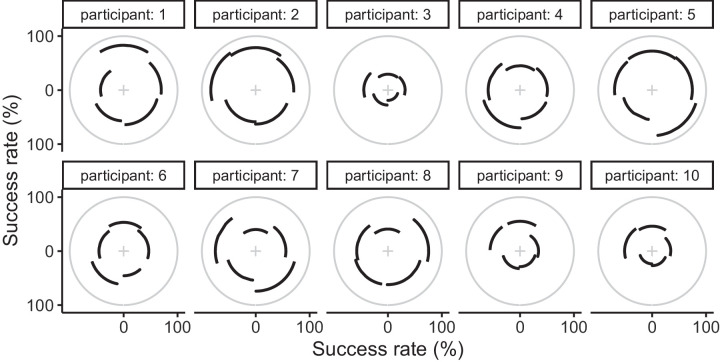
Participant-wise success rates by phase in the main task, aggregated for distinct phase bins (black line segments). Distance of the black lines from origin indicates the success rate per phase bin. Grey circles show a success rate of 100%. Based on the figure, there was no clear relationship between the phase where the target appeared and discrimination performance, but some variation in overall success rates between participants.

**Figure A5.**
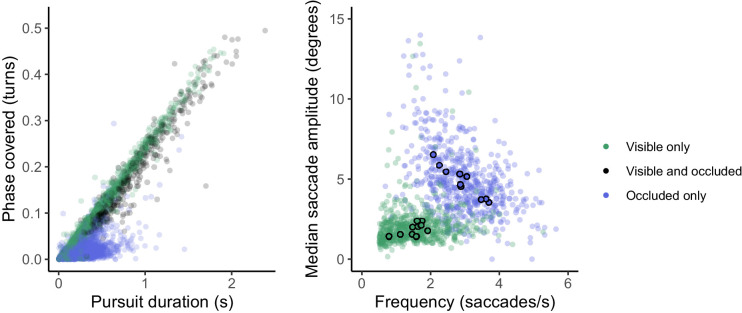
(Left) Pursuit/fixation duration plotted against the total phase covered by each segment, separately for the visible (green) and occluded (blue) periods. Segments spanning both periods are marked separately with black dots. The phase angle covered by pursuits during occlusion was relatively smaller than during visible periods, corresponding to the observed drop in pursuit gain. (Right) trial-wise frequency and median amplitude of saccades in each period. Black-bordered points represent participant medians. Both saccade frequency and amplitude were higher when the object was occluded than when it was visible. Larger (anticipatory) saccades during occlusion were linked to a smaller frequency of saccades (i.e., longer pursuit/fixation duration after the saccade).

**Figure A6.**
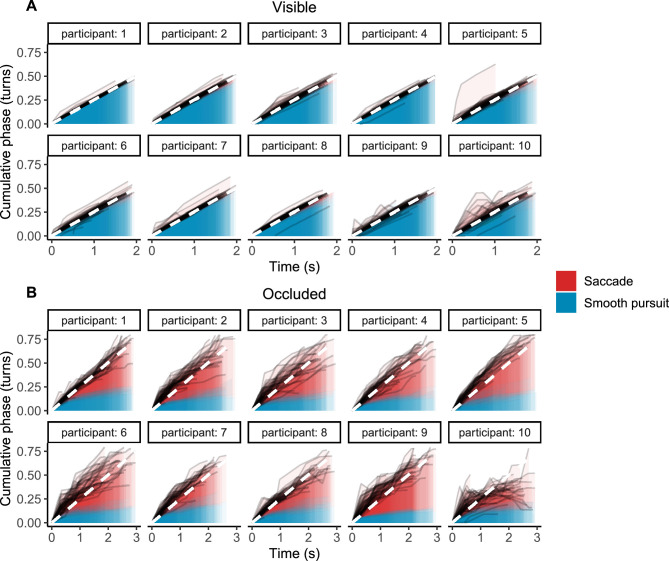
Participant-wise plots of cumulative phase covered by smooth pursuit (blue) and saccades (red) during visible (A) and occluded (B) periods. Dashed white line shows the phase of the tracked object.

## Footnotes

1 The geometry of the setup is such that the true horizontal FOV of the display is approximately 70°, but the linearization error at the circular path's radius is minimized by using an “effective FOV” of 78.8°, which we round to 80°.

## Appendix

### A.4 Phase and radius SD over time

Linear mixed models were fitted in R with package lme4 (Bates et al., 2014). To estimate effect sizes, we used marginal (variance explained by fixed factors) and conditional (variance explained by both fixed and random factors) *R^2^* values, following the approach by Nakagawa & Schielzeth (2013). See Table A1 for the results of two separate linear mixed models, where the dependent variable was either radius difference *SD* or phase difference *SD*; both models included time as a fixed independent variable, beginning from 0.5 seconds, and random effect of participant (intercept and slope). Both models used an unstructured covariance matrix. Note that participant 10 was excluded from these analyses owing to abnormal eye movement patterns.

**Table A1.** Results of the two linear mixed models, where the dependent variable was either phase difference *SD* (in turns) or radius difference *SD* (in degrees). In both models, the fixed independent variable was time (in seconds), starting from 0.5 seconds after occlusion onset. Note that the units of the dependent variables in the two models are different.

DV	Predictor	B	*SE*	t	Marginal *R^2^*	Conditional *R^2^*	Random effect *SD*	Residual *SD*
Phase difference *SD*					.35	.96		0.004
	Intercept	0.021	0.004	5.32			0.01	
	Time	0.025	0.004	6.16			0.01	
Radius difference *SD*					.05	.85		0.18
	Intercept	1.452	0.21	6.91			0.63	
	Time	0.213	0.14	1.49			0.43	
